# Global and local genetic diversity at two microsatellite loci in *Plasmodium vivax* parasites from Asia, Africa and South America

**DOI:** 10.1186/1475-2875-13-392

**Published:** 2014-10-02

**Authors:** Mette L Schousboe, Samir Ranjitkar, Rupika S Rajakaruna, Priyanie H Amerasinghe, Flemming Konradsen, Francisco Morales, Rosalynn Ord, Richard Pearce, Toby Leslie, Mark Rowland, Nahla Gadalla, Ib C Bygbjerg, Michael Alifrangis, Cally Roper

**Affiliations:** Centre for Medical Parasitology, Institute of International Health, Immunology, and Microbiology, University of Copenhagen, Copenhagen, Denmark; Department of Infectious Diseases, Copenhagen University Hospital, Copenhagen, Denmark; Department of Zoology, University of Peradeniya, Peradeniya, 20400 Sri Lanka; International Water Management Institute, Hyderabad, Andhra Pradesh India; Robles Y Pampite, Cymbaya, Universidad San Francisco de Quito, San Francisco de Quito, Ecuador; Faculty of Infectious and Tropical Diseases, London School of Hygiene and Tropical Medicine, Keppel Street, London, WC1 4HT UK

**Keywords:** Malaria, *Plasmodium vivax*, Genotyping, Microsatellites, Diversity, Heterozygosity

## Abstract

**Background:**

Even though *Plasmodium vivax* has the widest worldwide distribution of the human malaria species and imposes a serious impact on global public health, the investigation of genetic diversity in this species has been limited in comparison to *Plasmodium falciparum.* Markers of genetic diversity are vital to the evaluation of drug and vaccine efficacy, tracking of *P. vivax* outbreaks, and assessing geographical differentiation between parasite populations.

**Methods:**

The genetic diversity of eight *P. vivax* populations (n = 543) was investigated by using two microsatellites (MS), m1501 and m3502, chosen because of their seven and eight base-pair (bp) repeat lengths, respectively. These were compared with published data of the same loci from six other *P. vivax* populations.

**Results:**

In total, 1,440 *P. vivax* samples from 14 countries on three continents were compared. There was highest heterozygosity within Asian populations, where expected heterozygosity (H_e_) was 0.92-0.98, and alleles with a high repeat number were more common. Pairwise *F*_ST_ revealed significant differentiation between most *P. vivax* populations, with the highest divergence found between Asian and South American populations, yet the majority of the diversity (~89%) was found to exist within rather than between populations.

**Conclusions:**

The MS markers used were informative in both global and local *P. vivax* population comparisons and their seven and eight bp repeat length facilitated population comparison using data from independent studies. A complex spatial pattern of MS polymorphisms among global *P. vivax* populations was observed which has potential utility in future epidemiological studies of the *P. vivax* parasite.

**Electronic supplementary material:**

The online version of this article (doi:10.1186/1475-2875-13-392) contains supplementary material, which is available to authorized users.

## Background

Even though the malaria parasite *Plasmodium vivax* has the widest global distribution of the five human malaria species and imposes a serious impact on global public health, descriptions of global genetic diversity of this parasite are more limited than for *Plasmodium falciparum*. A recent study comparing the total genomic diversity among six *P. vivax* isolates drawn from Asia, Africa and South America with a comparable set of six *P. falciparum* isolates reported twice as much single nucleotide polymorphism (SNP) diversity and a far deeper divergence among the *P. vivax* geographic strains [1]. There are accumulating reports of clinically severe disease and drug-resistant *P. vivax* in some parts of the world [2–8], which reinforce the need for more detailed exploration of the global geography of *P. vivax* genetic diversity.

In the past, the most commonly used genetic markers for genotyping of *P. vivax* malaria parasites were antigen-coding genes, such as the gene encoding the circumsporozoite protein, *Pvcsp*, and the genes coding for the merozoite surface proteins *Pvmsp-1* and *Pvmsp-3α* [9–13]. Other antigen-coding genes such as *Pvgam-1, Pvdbp, Pvama-1* were used less frequently, possibly because of limited diversity of the individual loci [reviewed by [14]]. Comparison between populations using these markers has been complicated by differences in allele identification protocols used in different laboratories.

In recent years, microsatellites (MS) as short tandem repeats found throughout the *P. vivax* genome [15] have increasingly been used in studies of genetic diversity in *P. vivax*. As MS are generally non-coding, they are not subject to the same selective forces as antigen-encoding genes, and are thus more suitable for the analysis of *P. vivax* population structure [15, 16]. Commonly, multiple MS loci found on different chromosomes are used in studies of population structure [6, 17–20]. Repeat number polymorphism observed in a MS is the result of replication slippage that occurs during DNA replication, when the new strand mispairs with the template strand, and the degree of polymorphism is proportional to the underlying rate of mutation and the effective population size [15, 21]. Slippage events become more common as the total number of repeats increase [19]. Hence, short repeat arrays tend to be less polymorphic than longer repeat arrays [1, 22].

Most MS-based studies of genetic diversity and population structure of *P. vivax* parasites focus on one particular country or region, but three studies have compared population level diversity between countries or regions [17, 19, 20], with sample sets between 214 to 425 samples. Two of the studies use a 13–14 tri- and tetra-nucleotide repeat MS [19, 20], while the third study analyses nine MS with repeat lengths of two to eight base-pair (bp) [17]. All the studies have revealed high levels of microsatellite diversity, with few private alleles being unique to populations or a specific geographical area.

The amalgamation of data from independent studies analysing genetic diversity at MS is hindered by use of different MS markers and further compounded by difficulties in the standardization of fragment size estimation on different sequencer machines. To simplify comparison between studies, a uniform set of MS loci is needed. Selection of MS with repeat lengths above four bp possibly improves the repeatability of allele classification among different laboratories as has been observed for *P. falciparum* MS studies [16].

In the present study the genetic diversity of samples from Sri Lanka, Nepal, Pakistan, and Afghanistan in South Asia, Venezuela and Ecuador in South America and Sudan and Sao Tome in Africa were analysed, using two MS m1501 (with seven bp repeat lengths) and m3502 (with eight bp repeat lengths) located on chromosome 1 and 3, respectively, initially described by Imwong *et al*. [17]. The data generated were compared with the published allele frequency data from Korea [6], India, Laos, Thailand and Colombia [17] and PNG [18]. The study evaluates the global and local genetic diversity at these two MS loci and assesses their differentiation among *P. vivax* populations worldwide.

## Methods

### Sample collection

The current study analysed 543 *P. vivax* samples from Asia (Sri Lanka, Pakistan, Afghanistan, and Nepal), Africa (Sudan and São Tomé) and South America (Venezuela and Ecuador) and compared these data with published MS data from India, Thailand, Laos, Korea, PNG and Colombia described by others [6, 10, 17, 18]. In total, 1,440 samples from 14 countries across three continents were compared, and the details of where and when these were collected are detailed in Table 1.

**Table 1:** **Heterozygosity (He) at the two microsatellite loci m1501 and m3502 in every survey**

	Study site	n	Year	n (%)	m1501	MOI	n (%)	m3502	MOI	m1501 + m3502	Ref
					He			He		***n***(%)	
**Asia**	**Sri Lanka**	386	2002-2006	352 (91.2)	0.85	1.276	357 (92.5)	0.74	1.073	338 (87.6)	*and [10]
	**Nepal**	55	2006, 2009-2010	53 (96.4)	0.94	1.189	49 (89.1)	0.80	1.347	47 (85.5)	*and [23]
	**Pak/Afg**	329	2001, 2003-2007	315 (95.7)	0.91	1.365	314 (95.4)	0.80	1.650	309309 (93.9)	*and [24, 25]
	**Korea**	58	1996-2000, 2007	58 (100)	0.42	---	58 (100)	0.73	---	58 (100)	[6]
	**India**	90	2003-2004	78 (86.7)	0.90	---	79 (87.8)	0.86	---	---	[17]
	**Laos**	81	2001-2003	81 (100)	0.83	---	74 (91.4)	0.90	---	74 (91.4)	[17]
	**Thailand**	92	1992-1998	91 (98.9)	0.89	---	91 (98.9)	0.85	---	---	[17]
	**PNG**	108	2004-2005	107 (99.1)	0.91	1.907	108 (100)	0.86	1.648	107 (99.1)	[18]
**South A.**	**Columbia**	82	2001-2003	80 (97.6)	0.70	---	82 (100)	0.76	---	80 (97.6)	[17]
	**Venezuela**	130	1996-1997	113 (86.9)	0.73	1.416	98 (75.4)	0.57	1.235	97 (74.6)	*
	**Ecuador**	17	2009	17 (100)	0.22	1.176	17 (100.0)	0.76	1.176	17 (100.0)	*
**Africa**	**Sudan**	8	2006	7 (87.5)	0.71	1.571	4 (50.0)	1.00	1.235	3 (37.5)	*
	**São Tomé**	4	2000	4 (100.0)	0.83	1.250	4 (100.0)	0.83	2.250	4 (100.0)	*
	**n** ***=***	1440	1992-2010	1356 (94.2)		1.614	1335 (92.7)		1.490	1134/1258 (90.1)	

PNG: Papua New Guinea. Pak/Afg: Pakistan/Afghanistan. South A: South America. The number (and percentage) of samples successfully amplified for each MS locus and for the two loci combined are shown in every survey. Multiplicty of infection (MOI) is calculated by averaging the number of alleles detected in the total number of PCR positive samples. Data not available are indicated by “---”. Reference marked as * is this study.

### Samples from Nepal

Samples from Nepal were collected in two separate studies estimating the malaria burden in Nepal; 38 samples collected in 2009–2010 from Jhapa (n = 33) and Banke (n = 5) [23], and 17 samples in Kanchanpur (n = 5) and Jhapa (n = 12) in 2006 (Sean Hewitt, pers comm).

### Samples from Pakistan and Afghanistan

The samples collected between 2004 and 2006 from closely linked sites in Pakistan (n = 236) and Afghanistan (n = 93) 50 km apart were combined because of the similar demographic settings [24]. A second set of Pakistan samples (n = 139) was independently collected between 2004 and 2006 in Adizai, Baghicha, and Khagan villages, close to the North West Frontier Province Peshawar [25], while 60 additional samples were collected in the Ashaghroo refugee camp, Adizai, Pakistan (Kate Kolaczinski, pers comm).

### Samples from Ecuador

In total, 17 samples were collected in the Sucumbíos Province in Ecuador in 2009 as a part of the Malaria transmission and natural resource management in the Ecuadorian Amazon project (Francisco Morales, pers comm).

### Samples from Venezuela

The Venezuelan samples (n = 130) were from cross-sectional malaria surveys conducted in ten communities along the Padamo River, Amazonas State in 1996 and 1997 [12, 26].

### Samples from Africa and São Tomé

In Sudan, eight samples were collected in the village of Asar in Gedaref State during a community-based survey as a part of a *P. falciparum* artemether-lumefantrine efficacy trial during 2006 (Nahla Gadalla, pers comm). Four samples from São Tomé were collected in 2000 from Riboque (n = 3) and Porto Alegre (n = 1), kindly provided by Dr João Pinto.

### Samples from Sri Lanka

Samples from Sri Lanka were collected 2002–2006 from multiple sites across Sri Lanka (n = 386) [10].

### Published studies

There are published data on allelic polymorphism at the same MS loci in *P. vivax* populations in Colombia (n = 82), India (n = 90), Laos (n = 81), and Thailand (n = 92) previously described by Imwong *et al*. [17]. Other studies have described polymorphism in samples from Korea (n = 58) [6] and Papua New Guinea (PNG) (n = 108) [18]. The samples from PNG were used only to compare expected heterozygosity values (H_e_) as the frequency of the individual alleles is not published [19].

### Amplification and fragment analysis of the *Plasmodium vivax*samples

The 543 *P. vivax* samples analysed were available either as blood spots on filter paper, as DNA already extracted from filter paper (using the Chelex-100 method [27] with modifications detailed in [28]) or as a blood smear on glass slides. The blood smear samples were a subset of the Nepalese samples and the method of DNA extraction is described in [23]. Sample size of the individual locations, year of collection, number of amplified samples, and percentage of polyclonal samples per locus are shown in Table 1.

The two MS, m1501 and m3502, were amplified by a semi-nested PCR and analysed on an ABI 3730XL genetic analyzer (Applied Biosystems, Foster City, CA, USA) using primers described by Imwong *et al.* [17], using a procedure described previously [10]. The length of the PCR fragment was determined by reference to the Genescan 500 Liz size standard (Applied Biosystems), using Genemapper vs. 4.1 (Applied Biosystems). Repetition of PCR was performed with 2 μl DNA template in the primary PCR whenever a sample was negative at one of the loci. If a sample was PCR negative at both loci, the sample was excluded from further analysis. Limited volumes of DNA in some individual samples from Venezuela, São Tomé, Sudan, and Pakistan prevented repeat analysis of a subset of these samples.

Determining whether a sample was mono- or polyclonal was based on analysis of electropherograms obtained by Genemapper. Polyclonality of samples was determined when > one peak was seen in the electropherogram. The existence of more than one peak indicates the presence of multiple genotypes or clones within an infection. Multiplicity of infection (MOI) among a group of samples was calculated by dividing the total number of clones detected in all PCR-positive samples by the total number of PCR-positive samples.

For the estimation of allele frequencies one allele was counted per sample. This was to avoid oversampling of rare alleles. In the case of mixed infections, the ‘major’ allele was counted. The relative size of the peaks was used to establish major *versus* minor alleles; if one allele peak was twice the height of the other allele(s) then a major allele was assigned. If peaks were of equivalent size or minor peak greater than 50% of the major, the allele was chosen by computer randomization.

PCR fragment-length measurements were calibrated against known repeat number by sequencing a subset of 11 Pakistan samples on an ABI Prism 377 (Perkin-Elmer) using ExoSAP-IT PCR Clean-up Kit (GE Healthcare) and the Big Dye terminator reaction mix (Perkin-Elmer), and using the m1501 and m3502 primers. After sequencing, the individual haplotypes were aligned with the published sequence of the Sal-1 strain (GenBank accession number: AAKM01000015) and analysed by use of the DNASTAR-Lasergene software. There was good concordance between estimations of repeat number based on the PCR fragment size and that confirmed by direct sequencing.

### Measures of diversity and population differentiation

The analysis of allelic diversity measured in the *P. vivax* study populations used four tests: 1) expected heterozygosity (H_e_), which can be defined as the chance of drawing two different alleles from a population, ranging between 0–1. It was calculated as H_e_ = [n/(n-1)][1-∑pi^2^], where n is the number of samples and pi is the frequency of the i^th^ allele. The estimation of H_e_ for each of the two loci in *P. vivax* in PNG was taken from the original publication [18]; 2) computation of *F*_ST_ pairwise population genetic distances; 3) analysis of molecular variance (AMOVA) attributes the proportion of the total genetic variance which is found within populations (countries), between populations within groups (continents), and among groups; and, 4) isolation by distance (IBD) was used to test for any correlation between *F*_ST_ estimates and geographic distances using Mantel’s test [29]. The calculations were performed by plotting pairwise *F*_ST_/(1-*F*_ST_) against the natural logarithm of the geographical distance (in km). Evaluation of IBD was performed by showing R squared (R^2^), indicating the percentage of the variance explained by the model, the correlation coefficient *r* as a measure of the degree of linear relationship between two variables and the P-value. The calculations in all tests were performed by including both mono- and polyclonal samples.

The software used for the calculations of H_e_ was Excel add-in MS Toolkit® software, whereas the program FSTAT was used to perform the pairwise *F*_ST_ estimates and IBD calculations with 10,000 permutations [29]. Lastly, linkage equilibrium (LD) and AMOVA calculations were performed in Arlequin software version 3.11 [30], and significance of the AMOVA results was assessed by a randomization test with 10,000 permutations.

### Isolation by distance (IBD)

To enable calculations of IBD, geographical distances in km were determined by use of Google Earth [31]. The samples from India, Korea, Laos, Thailand, Ecuador, and Sudan were originally collected at one site in each country, while the remaining samples originated from two to ten sites per country. To simplify the IBD calculations, one site was chosen per country. In Sri Lanka, most samples were collected from the district of Anuradhapura, hence the site of Anuradhapura General Hospital was chosen. The majority from the Pakistan/Afghanistan sample collection was from the Peshawar district, hence this site was chosen. In Colombia, five sites were included in the study, with ten to 20 samples collected per site [17]. The Tumaco municipality is located approximately in the middle of the five districts, and so this was chosen to represent the Colombian samples. The Gedaref State in Sudan, Riboque in São Tomé, the Jhapa district in Nepal, the approximate middle of the DMZ between North and South Korea and the centre part of Rio Padamo (Venezuela) were chosen to represent these countries.

## Results

In total, 543 samples from Sri Lanka, Nepal, Pakistan and Afghanistan, Venezuela, Ecuador, Sudan and Sao Tome were analysed. The number and percentage of samples successfully amplified at each locus are shown in Table 1. The rate of success was similar for m1501 (94.2%) and m3502 (92.7%) (Table 1). Additional MS data on m1501 and m3502 obtained from published studies are also included in Table 1 and the H_e_ at each locus in every site is compared. A high H_e_ was observed at both loci in all the sites, with variations among sites being broadly consistent across the two loci. The South American sites had lower diversity (H_e_ range: m1501 = 0.22-0.73, m3502 = 0.57-0.76) than Asian (H_e_ range: m1501 = 0.42-0.94, m3502 = 0.73-0.90) and African sites (H_e_ range: m1501 = 0.71-0.83, m3502 = 0.83-1.00).

### Standardizing repeat number at microsatellites m1501 and m3502

For the analysis of genetic differentiation between populations, independently derived data from published studies of *P. vivax* in Thailand, Laos, India and Colombia published by Imwong *et al*. [17] and from Korea published by Honma *et al*. [6] were incorporated in the analysis. Allele nomenclature prevented amalgamation of data from a study in PNG [18] in the meta-analysis. To combine these data with the data obtained from the present study, fragment sizes were grouped according to the estimated number of repeats under a common allele name. Additional files 1 and 2 show how alleles common to different studies were matched and how the dataset was unified.

A few m1501 alleles from Sri Lanka and Pakistan samples and m3502 alleles in samples from Laos and Sri Lanka showed irregular spacing caused by a fragment size shift of a few nucleotides, presumably caused by insertion/deletion within the PCR amplified fragment. These irregular alleles were classified under the same repeat number as those of similar size elsewhere but are distinguished in the Tables by appending ‘a’ and ‘b’ to the repeat number. In the allele frequency and Fst calculations subgroups a and b were combined [see Additional files 1 and 2].

### Polymorphism at microsatellites m1501 and m3502

The m1501 marker discriminated 29 alleles in the global dataset while m3502 discriminated 18 alleles. The number of alleles differed remarkably between populations. Asian *P. vivax* populations were highly polymorphic at both loci, with the exception of the Korean *P. vivax* population. This contrasted with the South American *P. vivax* populations, which were less diverse. Diversity at m1501 was highest in Pakistan where 27 alleles were detected among 315 samples, while only two were seen in Ecuador (n = 17) and three in Korea (n = 58) [see Additional file 1]. For the m3502 marker, again, the highest diversity was found in Pakistan, while the lowest diversity was observed in the African and Korean samples where only three alleles and four alleles per site were detected, respectively [see Additional file 2]. For both markers, the most common alleles had a repeat size ranging from three to six repeats. This was true among all study populations except Korea, where a 13-repeat m3502 allele, named 216, comprised 39.7% of the 58 samples.

**Figure 1:**
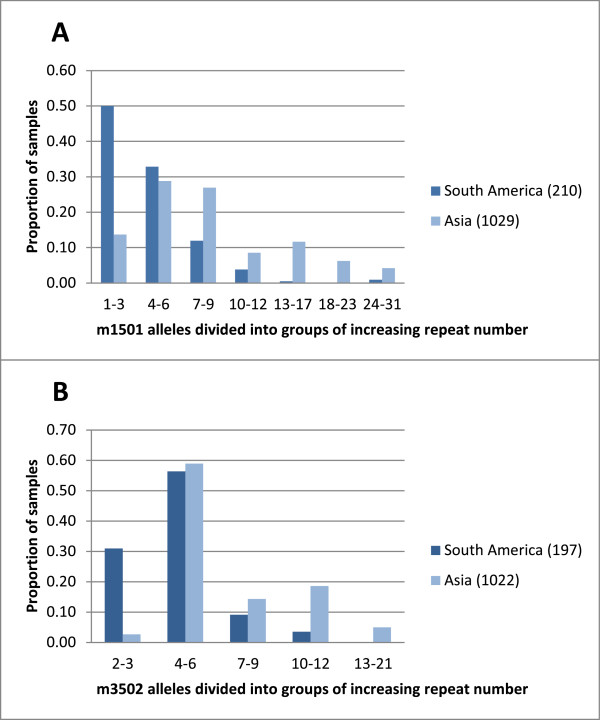
**Allelic diversity at the MS m1501 (A) and m3502 (B) in** ***P. vivax*** **samples from South America (Columbia, Venezuela and Ecuador) and Asia (India, Laos, Thailand, Korea, Nepal, Pakistan and Sri Lanka).** The MS alleles are divided into groups of increasing fragment size according to the number of repeats. In brackets are mentioned the number of samples included from each continent.

Alleles with long fragment lengths, above 12 repeats for m1501 and 10 repeats for m3502, were detected more commonly in Asian than elsewhere (Figure 1A and B). In general, the maximum number of repeats seen in South America and Africa was 17 repeats for m1501 and 11 repeats for m3502 repeats while in Asia, repeat lengths of up to 31 for m1501 and 19 for m3502 were common. An exception to this trend was two samples from Venezuela where long-fragment m1501 alleles of 31 repeats were observed, while a 21-repeat m3502 allele was detected in Sudan.

### Pairwise differentiation between *Plasmodium vivax*populations

*Plasmodium vivax* populations in South America (Venezuela, Colombia and Ecuador) showed evidence of population structure. *F*_ST_ using the m1501 marker revealed a significant difference between Colombia and the two other South American countries (*F*_ST_ = 0.05-0.21), while *F*_ST_ using the m3502 marker, found significant differences between Venezuela and the two other South American countries (*F*_ST_ = 0.26-0.27) [see Additional file 3]. Generally, *P. vivax* populations from these three countries were significantly different from all the Asian countries, with few exceptions. Low, but significant pairwise *F*_ST_ estimates were found among the Asian countries; 0.02-0.31 (m1501) and 0.02-0.14 (m3502). The analysis of IBD was done by using pairwise genetic and geographic distances. Evidence for IBD was found for both loci by Mantel’s test; m1501: (R^2^ = 6.35, r = 0.0789, P = 0.04), and m3502: (R^2^ = 7.99, r = 0.0285, P = 0.02).

*F*_ST_ estimates using the two loci combined were slightly higher and pairwise estimates between *P. vivax* populations from Korea, Nepal, Pakistan, Sri Lanka, Venezuela, Ecuador, Sudan, and São Tomé, revealed *F*_ST_ between 0.01-0.40 [see Additional file 4]. The highest difference was found between the *P. vivax* populations in Ecuador and Sudan and the lowest between *P. vivax* populations from Nepal and Sri Lanka. The two-locus haplotypes were often not specific to a single sample set from one country, but present in more than one *P. vivax* population as for instance the ‘107-142’ haplotype seen in Nepal, Pakistan and Sri Lanka [see Additional file 5].

### The allelic diversity within and between *Plasmodium vivax*populations

The allelic diversity within the different *P. vivax* populations was estimated by H_e_. The average H_e_ per locus was 0.90 (m1501) and 0.84 (m3502). The H_e_ varied considerably from site to site, but was generally lowest in the three South American countries (H_e_ = 0.22-0.76) and highest in the Asian populations (H_e_ = 0.80-0.94) with the exception of Korea, which possessed very low H_e_ values of 0.42 (m1501) and 0.73 (m3502) (Table 1 and Additional file 6). The AMOVA analysis found the bulk (~89%) of genetic variance occurs within the 14 *P. vivax* populations [see Additional file 7], ~9% of the variance occurs between populations within continents, and only about 4% of the variance occurs between the continents [see Additional file 7].

Comparison of the three continents (South America, Asia and Africa) showed that the South American *P. vivax* population was distinct from the two other continents, with low but significant *F*_ST_ estimates (m1501 = 0.09-0.18, m3502 = 0.08-0.11) (Table 2). This may be attributable to the lower diversity found in South America where the m1501 marker possessed the lowest H_e_ values (n = 210, H_e_ = 0.71), thereafter Africa (n = 11, H_e_ = 0.84) whereas Asia had the most diverse *P. vivax* populations in this study (n = 1,028, H_e_ = 0.92). The same ranking was seen using the m3502 marker: South America (n = 197, H_e_ = 0.76), Africa (n = 8, H_e_ = 0.86) and Asia (n = 1,022, H_e_ = 0.84).

**Table 2:** **Genetic differentiation between** ***P. vivax*** **populations measured by pairwise** ***F***_**ST**_

m1501	Africa	South America	Asia
Africa (11)		**	NS
South America (210)	0.1824		**
Asia (1028)	0.0446	0.0869	
**m3502**	**Africa**	**South America**	**Asia**
Africa (8)		**	NS
South America (197)	0.1050		**
Asia (1022)	0.0049	0.0752	

The pairwise significance after standard Bonferroni corrections are listed as: “**” significance at the 1% nominal level while “NS” stands for non-significant.

## Discussion

The main objective of the current study was to evaluate the global genetic diversity of *P. vivax* populations by examining the two MS, m1501 and m3502 in *P. vivax* samples collected from Ecuador, Venezuela, Sri Lanka, Afghanistan, Pakistan, Nepal, São Tomé, and Sudan (n = 543). Although some of these counties were represented by a large *P. vivax* sample set, the limited available sample set from Ecuador (n = 17), São Tomé (n = 4) and Sudan (n = 8) means caution should be applied to the interpretation of the results regarding these sites. Furthermore, there is a significant time interval between the collection of the Venezuelan samples in 1996–1997 and the other South American samples some years later. Where possible, the resulting data were compared with published data from studies of the same markers in Colombia, India, Laos, Thailand, PNG, and Korea (n = 897).

The geographical genetic diversity of the *P. vivax* populations was shown to be highly diverse, with the majority of the diversity found to be present within the populations (~89%). In total, 29 m1501 and 18 m3502 alleles were detected with average H_e_ estimates per allele of 0.90 (m1501) and 0.84 (m3502), lowest in the South American samples and highest in Asia. The H_e_ values found in this study by use of only two MS (H_e_ = 0.78-0.98) were high compared to H_e_ estimates reported by other studies (H_e_ = 0.48-0.87), which have analysed samples from the same three continents including nine to 14 MS [6, 17, 20, 32], and the H_e_ estimates of 0.26 to 0.91 reviewed by others [14].

South American and Asian populations were found to be significantly different from each other by pairwise *F*_ST_, whereas the African population generally could not be distinguished statistically from most other populations, most probably this was due to the limited sample size. The *F*_ST_ estimates ranged from 0.02-0.63 (m1501) and 0.03-0.28 (m3502) per site, and a Mantel test correlating pairwise genetic and geographic distances between populations showed evidence for IBD.

The high frequency of alleles with low repeat number (one to six repeats) was common for all the *P. vivax* populations, and the rarer long-repeat fragments were mainly restricted to Asia. Only a limited number of samples from Africa were analysed, but these suggest Africa is intermediate between South American and Asian populations. The long repeat-alleles appearing in the Asian *P. vivax* populations might relate to factors, such as higher *P. vivax* endemicity in Asian countries.

Malaria endemicity is expected to have a significant influence on genetic diversity and levels of inbreeding/outbreeding. Among the populations sampled in the present study, Asian populations were the most diverse, although a range of H_e_ estimates and MOI values were measured among the individual populations sampled. The maximum MOI for an individual sample was six clones and this was found in Pakistan. The mean MOI in Pakistan was 1.55 and rates of heterozygosity were also high. The complexity of the Pakistan/Afghanistan population is striking, since these areas are not generally considered highly endemic. Similar rates of heterozygosity were reported in PNG [33] and the equivalent mean MOI value there was 1.82. It is generally accepted that high polyclonality increases the probability for heterogametic genetic recombination during the sexual cycle occurring in the mosquitoes, resulting in sporozoites with novel genotypes [34]. This may also promote heterozygosity at MS loci through a process of strand slippage during recombination. The mutation rate of MS in the *P. vivax* genome is unknown, but the high rates of complexity observed here are fully consistent with that reported from previous studies of *P. vivax* [10, 32, 35].

The above model of polyclonality and diversity is developed based on extensive and cumulative *P. falciparum* research, and is not the complete explanation of the mechanisms that contribute to *P. vivax* diversity. Genome wide analyses of MS diversity have shown *P. vivax* diversity is significantly greater than *P. falciparum* [1], but also that genome-wide SNP diversity is greater among *P. vivax* than *P. falciparum* [1]. The same study showed a longer time to most recent common ancestor among *P. vivax* isolates suggesting *P. vivax* diversity is more ancient. Although just six *P. vivax* isolates were compared, similar geographic trends were observed. South American isolates Brazil 1, Salvador 1 and Peru shared a more recent common ancestor with each other than with isolates from Asia (India, Korea) and Africa (Mauritania), which had deep branch lengths and did not cluster. The data presented in this study also showed high level of diversity and differentiation between the South American and Asian populations and the findings indicate that further investigation of genome-wide diversity among *P. vivax* populations from Central and Southeast Asia may reveal even greater levels of genetic diversity.

A ‘*P. falciparum*-model’ may not entirely predict and explain genetic diversity of *P. vivax* populations. Important biological differences between *P. vivax* and *P. falciparum* may also be at play. In *P. vivax*, gametocytogenesis occurs earlier in clinical episodes, and reticulocytes are essential to both invasion and relapse [36]. Significant linkage disequilibrium has been observed in the included sample set from Sri Lanka [10], the Colombian samples [17], the Korean samples [6], and by others analysing samples from Sri Lanka [19] and Brazil [35] and these findings suggest that clonal expansions of specific genotypes may be epidemiologically significant and further studies are needed to elucidate the local dynamics of *P. vivax* epidemics.

## Conclusions

The data presented in this study show the utility of the MS m1501 and m3502 in studies of *P. vivax* population structure, irrespective of geographical origin, although more markers might be needed in hypo-endemic areas if distinguishing between individual parasites is a priority. Ultimately, whole- genome analyses will provide detailed estimation of the total genetic diversity –the results presented in this study suggests that the greatest diversity will be in Southeast Asia.

### Ethical statement

Clearance for analysis of *Plasmodium* genes were approved by London School of Hygiene Tropical Medicine and Hygiene Ethics Board, locally by Bioethics Committee, Pakistan Medical Research Council and Directorate of Public Health, Jalalabad, Nangahar, Comitee de Bioetica Universidad San Francisco de Quito and the Nepal Health Research Council.

## Electronic supplementary material

Additional file 1: **Allelic diversity at the m1501 locus of** ***P. vivax*** **samples obtained from various malaria endemic countries.** (PDF 11 KB)

Additional file 2: **Allelic diversity at the m3502 locus of** ***P. vivax*** **samples obtained from various endemic countries.** (PDF 19 KB)

Additional file 3: **Population differentiation at m1501 and m3502 allelic estimated by pairwise fixation index, F**_**ST**_ **.** (PDF 13 KB)

Additional file 4: **Population differentiation estimated by pairwise fixation index, F**_**ST**_ **using the combined MS data from m1501 and m3502.** (DOCX 16 KB)

Additional file 5: **Allelic diversity for the combined MS genotype “m1501-3502” haplotypes per study site and in total.** (DOCX 18 KB)

Additional file 6: **Expected Heterozygosity (He) two** ***P. vivax*** **microsatellites, m1501 and 3502 in each site.** (DOCX 15 KB)

Additional file 7: **Global analysis of molecular variance (AMOVA) among** ***P. vivax*** **populations and continents (groups).** (DOCX 16 KB)
