# Supplementary material for: Global and local genetic diversity at two microsatellite loci in Plasmodium vivax parasites from Asia, Africa and South America
Source: Malar J. 2014 Oct 2;13:392. doi: 10.1186/1475-2875-13-392 (PMC4200131; doi:10.1186/1475-2875-13-392)
Supplement: Supplementary file 1 — Additional file 1: Allelic diversity at the m1501 locus of P. vivax samples obtained from various malaria endemic countries. (PDF 11 KB) [file 12936_2014_3558_MOESM1_ESM.pdf]

Additional file 1

Title: **Allelic diversity at the m1501 locus of *P. vivax* samples obtained from various malaria endemic countries**

Description: Data from 3 independent studies have been combined by grouping equivalent fragment sizes under a common allele name. The first column shows the allele name, designated by the number of 8bp repeats with predicted fragment size based on the Sal 1 reference sequence (GenBank accession number AAKM01000015). Surveys analysed in this study are from Sri Lanka Venezuela, Ecuador, Nepal, Pakistan, Afghanistan, Sudan and São Tomé whereas surveys described in [17] are from Columbia, India, Laos and Thailand. The Korean survey described in [6] fragment sizes determined by sequencing was applied and not fragment length measurement as the other samples. The number of samples in which each allele was detected is indicated in brackets. Some of the repeat lengths are of 2-3bp difference as compared to the predicted fragment size; these have been divided into group "a" and "b" groups but still defined within the same repeat length.

**Allele Name**	**Allele size classification**		**The Americas**			**Asia**							**Africa**	
**Number of 8 bp repeats (predicted fragment size (bp))**	**This study**	***Imwong et al.*[17]**	**Columbia Size (n)**	**Venezuela Size (n)**	**Ecuador Size (n)**	**India Size (n)**	**Laos Size (n)**	**Thailand Size (n)**	**Korea Size (n)**	**Nepal Size(n)**	**Pakistan Size(n)**	**Sri Lanka Size(n)**	**Sudan Size(n)**	**São Tomé Size(n)**
1 (71)	74		---	---	---	---	---	---	---	---	74 (3)	---	---	---
2 (78)	78	76	---	78 (3)	---	---	---	76 (1)	---	78 (3)	78 (37)	78 (1)	---	---
3 (85)	86	83	83 (33)	86 (54)	86 (15)	83 (1)	83 (11)	83 (12)	~86 (42)	86 (4)	86 (25)	86 (1)	---	---
4 (92)	92	90	90 (1)	92 (3)	---	---	90 (12)	90 (11)	---	92 (1)	92 (6)	92 (1)	---	---
5 (99)	100	97	97 (14)	100 (14)	---	97 (4)	97 (25)	97 (19)	~99 (15)	100 (10)	100 (44)	100 (13)	100 (1)	100 (1)
6 (106)	107	104	104 (26)	107 (11)	---	104 (16)	104 (13)	104 (11)	---	107 (6)	107 (47)	107 (42)	107 (1)	107 (1)
7 (113)	114	111	111 (6)	114 (3)	---	111 (6)	111 (8)	111 (9)	---	114 (3)	114 (34)	114 (7)	114 (4)	---
8 (120)	121	118	---	---	---	118 (10)	118 (3)	118 (6)	---	121 (4)	121 (15)	---	---	---
9a (127)	124	125	---	124 (2)	---	125 (9)	125 (4)	125 (11)	---	---	---	---	---	---
9b (127)	128	---	---	128 (14)		---	---	---	---	128 (2)	128 (31)	128 (115)		
10 (134)	135	132	---	---	135 (2)	132 (7)	132 (3)	132 (3)	---	135 (2)	135 (12)	---	---	---
11a (141)	139	139	---	---	---	139 (4)	139 (1)	139 (2)	---	---	139 (1)	139 (4)	---	---
11b (141)	142	---	---	142 (4)	---	---	---	---	~141 (1)	142 (2)	142 (4)	142 (1)	---	---
12 (148)	150	150	---	150 (2)	---	150 (5)	---	150 (2)	---	150 (2)	150 (6)	150 (26)	150 (1)	---
13 (155)	158	156	---	---	---	156 (5)	---	156 (1)	---	158 (2)	158 (8)	158 (5)	---	---
14 (162)	165	163	---	---	---	163 (6)	---	---	---	165 (2)	165 (3)	165 (10)	---	165 (2)
15 (169)	172	171	---	---	---	171 (3)	---	---	---	---	172 (9)	172 (4)	---	---
16 (176)	179	---	---	---	---	---	---	---	---	179 (3)	179 (1)	179 (25)	---	---
17 (183)	184	185	---	184 (1)	---	185 (2)	---	185 (1)	---	184 (2)	184 (15)	184 (12)	---	---
18 (190)	192	191	---	---	---	---	---	191 (1)	---	---	---	192 (3)	---	---
19 (197)	199	195	---	---	---	---	195 (1)	195 (1)	---	199 (1)	199 (1)	199 (5)	---	---
20 (204)	206	---	---	---	---	---	---	---	---	---	206 (2)	206 (8)	---	---
21 (211)	214	---	---	---	---	---	---	---	---	214 (1)	214 (1)	214 (2)	---	---
22 (218)	220	---	---	---	---	---	---	---	---	---	---	220 (5)	---	---
23 (225)	227	---	---	---	---	---	---	---	---	---	227 (1)	227 (31)	---	---
24 (232)	234	---	---	---	---	---	---	---	---	---	234 (1)	---	---	---
25 (239)	241	---	---	---	---	---	---	---	---	---	241 (2)	241 (25)	---	---
26 (246)	248	---	---	---	---	---	---	---	---	---	---	248 (5)	---	---
27 (253)	255	---	---	---	---	---	---	---	---	---	255 (2)	---	---	---
28 (260)	262	---	---	---	---	---	---	---	---	262 (1)	262 (2)	---	---	---
31 (281)	284	---	---	284 (2)	---	---	---	---	---	284 (2)	284 (2)	284 (1)	---	---
**n=**			80	113	17	78	81	91	58	53	315	352	7	4
