# Supplementary material for: Global and local genetic diversity at two microsatellite loci in Plasmodium vivax parasites from Asia, Africa and South America
Source: Malar J. 2014 Oct 2;13:392. doi: 10.1186/1475-2875-13-392 (PMC4200131; doi:10.1186/1475-2875-13-392)
Supplement: Supplementary file 2 — Additional file 2: Allelic diversity at the m3502 locus of P. vivax samples obtained from various endemic countries. (PDF 19 KB) [file 12936_2014_3558_MOESM2_ESM.pdf]

Additional file 2

Title: **Allelic diversity at the m3502 locus of *P. vivax* samples obtained from various endemic countries.**

Description: Data from 3 independent studies have been combined by grouping equivalent fragment sizes under a common allele name. The first column shows the allele name, designated by the number of 8bp repeats, with predicted fragment size based on the Sal 1 reference sequence (GenBank accession number AAKM01000015). Surveys analysed in this study are from Sri Lanka Venezuela, Ecuador, Nepal, Pakistan, Afghanistan, Sudan and São Tomé whereas surveys described in [17] are from Columbia, India, Laos and Thailand. The Korean survey described in [6] fragment sizes determined by sequencing was applied and not fragment length measurement as the other samples. The number of samples in which each allele was detected is indicated in brackets. Some of the repeat lengths are of 2-3bp difference as compared to the predicted fragment size; these have been divided into group “a” and “b” groups but still defined within the same repeat length.

**Sal 1 (m3502)**	**Alleles**		**The Americas**			**Asia**							**Africa**	
**Number of 8 bp repeats (predicted fragment size (bp))**	**This study**	**Imwong *et al.* [17]**	**Columbia Size (n)**	**Venezuela Size (n)**	**Ecuador Size (n)**	**India Size (n)**	**Laos Size (n)**	**Thailand Size (n)**	**Korea Size (n)**	**Nepal Size (n)**	**Pakistan Size (n)**	**Sri Lanka Size (n)**	**Sudan Size (n)**	**São Tomé Size (n)**
2 (128)	---	128	---	---	---	---	128 (1)	---	---	---	---	---	---	---
3 (136)	133	136	136 (32)	133 (22)	133 (7)	136 (10)	136 (5)	---	---	133 (2)	133 (9)	---	133 (1)	---
4a (144)	142	144	144 (8)	142 (60)	142 (1)	144 (15)	144 (13)	144 (15)	~ 144 (16)	142 (14)	142 (111)	142 (18)	---	---
4b (144)	145	---	---	---	---	---	---	---	---	---	---	145 (1)	---	---
5a (152)	---	150	150 (22)	---	---	---	150 (8)	---	---	---	---	---	---	---
5b (152)	151	152	---	151 (5)	151 (1)	152 (19)	152 (7)	152 (17)	~ 152 (10)	151 (16)	151 (58)	151 (158)	151 (1)	151 (2)
6a (160)	---	158	158 (8)	---	---	---	158 (5)	---	---	---	---	---	---	---
6b (160)	159	161	---	159 (6)	---	161 (6)	---	161 (25)	---	159 (4)	159 (39)	159 (31)	159 (1)	159 (1)
7a (168)	167	169	---	167 (1)	167 (4)	169 (13)	---	169 (10)	---	167 (4)	167 (9)	167 (42)	---	---
7b (168)	---	172	172 (4)	---	---	---	172 (14)	---	---	---	---	---	---	---
8a (176)	175	178	---	---	175 (4)	178 (5)	---	178 (6)	---	175 (4)	175 (19)	---	---	---
8b (176)	---	180	180 (5)	---	---	---	180 (4)	---	---	---	---	---	---	---
9a (184)	183	185	---	---	---	185 (1)	---	185 (3)	---	---	183 (7)	---	---	---
9b (184)	---	188	---	---	---	---	188 (6)	---	---	---	---	---	---	---
10a (192)	191	194	---	191 (2)	---	194 (6)	---	194 (6)	---	---	191 (36)	191 (14)	---	---
10b (192)	---	196	196 (3)	---	---	---	196 (6)	---	---	---	---	---	---	---
11a (200)	199	201	---	199 (2)	---	201 (2)	---	201 (5)	~ 200 (9)	199 (4)	199 (24)	199 (72)	---	199 (1)
11b (200)	---	204	---	---	---	---	204 (1)	---	---	---	---	---	---	---
12a (208)	206	208	---	---	---	208 (2)	---	---	---	206 (1)	---	---	---	---
12b (208)	---	212	---	---	---	---	212 (3)	---	---	---	---	---	---	---
13 (216)	215	---	---	---	---	---	---	---	~ 216 (23)	---	---	215 (21)	---	---
14 (224)	---	228	---	---	---	---	228 (1)	---	---	---	---	---	---	---
15 (232)	231	---	---	---	---	---	---	---	---	---	231 (2)	---	---	---
16 (240)	---	241	---	---	---	---	---	241 (2)	---	---	---	---	---	---
17 (248)	---	249	---	---	---	---	---	249 (1)	---	---	---	---	---	---
19 (264)	---	265	---	---	---	---	---	265 (1)	---	---	---	---	---	---
21 (280)	279	---	---	---	---	---	---	---	---	---	---	---	279 (1)	---
**n** =			82	98	17	79	74	91	58	49	314	357	4	4
