# Supplementary material for: Global and local genetic diversity at two microsatellite loci in Plasmodium vivax parasites from Asia, Africa and South America
Source: Malar J. 2014 Oct 2;13:392. doi: 10.1186/1475-2875-13-392 (PMC4200131; doi:10.1186/1475-2875-13-392)
Supplement: Supplementary file 3 — Additional file 3: Population differentiation at m1501 and m3502 allelic estimated by pairwise fixation index, F ST . (PDF 13 KB) [file 12936_2014_3558_MOESM3_ESM.pdf]

Additional file 3

Title: **Population differentiation at m1501 and m3502 allelic estimated by pairwise fixation index, F_{ST}.**

Description: The pairwise significance after standard Bonferroni corrections are listed as: "***"significance at the 0.1% nominal level, "**" significance at the 1% nominal level and "*" significance at the 5% nominal level, while "NS" stands for non-significant.

m1501 (n)	Columbia	Ecuador	Venezuela	India	Korea	Laos	Nepal	Pakistan	Sri Lanka	Thailand	São Tomé	Sudan
**Columbia (80)***		***	***	***	***	***	***	***	***	***	NS	NS
**Ecuador (17)**	0.2125		NS	***	NS	***	***	***	***	***	*	**
**Venezuela (113)**	0.0428	0.1166		***	***	***	***	***	***	***	NS	NS
**India (78)***	0.1239	0.3371	0.1406		***	***	NS	***	***	***	NS	NS
**Korea (58)****	0.1464	0.0944	0.0675	0.3125		***	***	***	***	***	**	***
**Laos (81)***	0.0766	0.3137	0.0990	0.0633	0.2292		*	***	***	NS	NS	NS
**Nepal (53)***	0.0912	0.3049	0.0962	0.0172	0.2497	0.0163		NS	***	NS	NS	NS
**Pakistan (315)**	0.0856	0.2796	0.0919	0.0199	0.2229	0.0303	0.0010		***	*	NS	NS
**Sri Lanka (352)**	0.1766	0.3494	0.1506	0.0512	0.3072	0.1119	0.0670	0.0589		***	NS	NS
**Thailand (91)***	0.0789	0.2820	0.0771	0.0326	0.2185	-0.0005	0.0046	0.0118	0.0680		NS	NS
**São Tomé (4)**	0.1528	0.6276	0.1906	0.0219	0.4522	0.0557	0.0099	0.0434	0.1062	0.0536		NS
**Sudan (7)**	0.2064	0.6094	0.2353	0.0905	0.4844	0.1029	0.0809	0.0697	0.1605	0.0840	0.1767	

m3502 (n)	Columbia	Ecuador	Venezuela	India	Korea	Laos	Nepal	Pakistan	Sri Lanka	Thailand	São Tomé	Sudan
**Columbia (82)***		***	***	***	***	***	***	***	***	***	***	*
**Ecuador (17)**	0.0969		***	NS	***	***	*	***	***	***	NS	NS
**Venezuela (98)**	0.2728	0.2552		***	***	***	***	***	***	***	NS	NS
**India (79)***	0.1336	0.0776	0.2524		***	***	***	***	***	*	NS	NS
**Korea (58)****	0.2368	0.2539	0.3502	0.1215		***	***	***	***	***	NS	**
**Laos (74)***	0.0860	0.1349	0.2560	0.0623	0.1306		***	***	***	***	NS	NS
**Nepal (49)***	0.2097	0.1411	0.1412	0.0677	0.1819	0.1205		NS	***	***	NS	NS
**Pakistan (314)**	0.2074	0.1613	0.0819	0.1028	0.1948	0.1374	0.0154		***	***	NS	NS
**Sri Lanka (357)**	0.2541	0.2108	0.2849	0.0766	0.1573	0.1563	0.0517	0.1116		***	NS	*
**Thailand (91)***	0.1841	0.1472	0.2699	0.0256	0.1377	0.0859	0.0827	0.0969	0.0905		NS	NS
**São Tomé (4)**	0.2187	0.1921	0.3269	0.0075	0.1292	0.0817	-0.0209	0.0507	-0.0989	-0.0207		NS
**Sudan (4)**	0.0638	0.0341	0.2358	-0.0262	0.1464	0.0249	0.0141	0.0455	0.0415	-0.0239	-0.1282	
