# Supplementary material for: Global and local genetic diversity at two microsatellite loci in Plasmodium vivax parasites from Asia, Africa and South America
Source: Malar J. 2014 Oct 2;13:392. doi: 10.1186/1475-2875-13-392 (PMC4200131; doi:10.1186/1475-2875-13-392)
Supplement: Supplementary file 4 — Additional file 4: Population differentiation estimated by pairwise fixation index, F ST using the combined MS data from m1501 and m3502. (DOCX 16 KB) [file 12936_2014_3558_MOESM4_ESM.docx]

**Additional file 4**

Title: **Population differentiation estimated by pairwise fixation index, F_ST_ using the combined MS data from m1501 and m3502**

Description: In brackets are mentioned number of positive m1501 samples and m3502 samples respectively. The pairwise significance after standard Bonferroni corrections are listed as: “***”significance at the 0.1% nominal level, “**” significance at the 1% nominal level and “*” significance at the 5% nominal level, while “NS” stands for non-significant.

| m1501-3502 | Ecuador | Venezuela | Korea | Nepal | Pakistan | Sri Lanka | São Tomé | Sudan |
| --- | --- | --- | --- | --- | --- | --- | --- | --- |
| Ecuador (17,17) |  | *** | *** | *** | *** | *** | ** | * |
| Venezuela (113,98) | 0.1880 |  | *** | *** | *** | *** | * | * |
| Korea (56, 56) | 0.2084 | 0.2526 |  | *** | *** | *** | *** | ** |
| Nepal (53,49) | 0.2305 | 0.1170 | 0.2619 |  | NS | *** | NS | NS |
| Pakistan (315,314) | 0.2276 | 0.0873 | 0.2384 | 0.0078 |  | *** | NS | NS |
| Sri Lanka (352,357) | 0.2906 | 0.2181 | 0.2916 | 0.0599 | 0.0842 |  | NS | *** |
| São Tomé (4,4) | 0.3977 | 0.2570 | 0.3602 | -0.0041 | 0.0468 | 0.0215 |  | NS |
| Sudan (7,4) | 0.3360 | 0.2355 | 0.3445 | 0.0506 | 0.0585 | 0.1091 | 0.0334 |  |
