# Supplementary material for: Global and local genetic diversity at two microsatellite loci in Plasmodium vivax parasites from Asia, Africa and South America
Source: Malar J. 2014 Oct 2;13:392. doi: 10.1186/1475-2875-13-392 (PMC4200131; doi:10.1186/1475-2875-13-392)
Supplement: Supplementary file 5 — Additional file 5: Allelic diversity for the combined MS genotype “m1501-3502” haplotypes per study site and in total. (DOCX 18 KB) [file 12936_2014_3558_MOESM5_ESM.docx]

**Additional file 5**

Title: **Allelic diversity for the combined MS genotype “m1501-3502” haplotypes per study site and in total.**

Description: Only genotypes with counts above 10 are included in the table.

| 1501-3502 | Nepal | Pakistan | Sri Lanka | Ecuador | Venezuela | Sudan | São Tomé | N |
| --- | --- | --- | --- | --- | --- | --- | --- | --- |
| 128-151 | --- | 6 | 83 | --- | 1 | --- | --- | 90 |
| 107-142 | 2 | 25 | 2 | --- | 7 | --- | --- | 36 |
| 86-142 | --- | 4 | --- | 1 | 23 | --- | --- | 28 |
| 107-151 | 3 | 7 | 15 | --- | --- | --- | --- | 25 |
| 241-199 | --- | 1 | 24 | --- | --- | --- | --- | 25 |
| 78-142 | 1 | 22 | --- | --- | --- | --- | --- | 23 |
| 100-151 | 2 | 6 | 11 | --- | --- | --- | 1 | 20 |
| 179-159 | 1 | --- | 19 | --- | --- | --- | --- | 20 |
| 107-167 | --- | 2 | 17 | --- | --- | --- | --- | 19 |
| 114-142 | 2 | 11 | 1 | --- | 3 | --- | --- | 17 |
| 100-142 | 3 | 9 | --- | --- | 4 | --- | --- | 16 |
| 128-142 | --- | 6 | 1 | --- | 8 | --- | --- | 15 |
| 128-199 | --- | 2 | 12 | --- | 1 | --- | --- | 15 |
| 150-206 | --- | --- | 15 | --- | --- | --- | --- | 15 |
| 227-199 | --- | --- | 15 | --- | --- | --- | --- | 15 |
| 128-191 | --- | 6 | 8 | --- | --- | --- | --- | 14 |
| 227-151 | --- | --- | 14 | --- | --- | --- | --- | 14 |
| 184-167 | 1 | --- | 10 | --- | --- | --- | --- | 11 |
| n/alleles total | 33/47 | 101/302 | 63/336 | 5/15 | 17/64 | 3/3 | 4/4 | 146/770 |
