# Supplementary material for: Global and local genetic diversity at two microsatellite loci in Plasmodium vivax parasites from Asia, Africa and South America
Source: Malar J. 2014 Oct 2;13:392. doi: 10.1186/1475-2875-13-392 (PMC4200131; doi:10.1186/1475-2875-13-392)
Supplement: Supplementary file 6 — Additional file 6: Expected Heterozygosity (He) two P. vivax microsatellites, m1501 and 3502 in each site. (DOCX 15 KB) [file 12936_2014_3558_MOESM6_ESM.docx]

**Additional file 6**

Title: **Expected Heterozygosity (He) two *P. vivax* microsatellites, m1501 and 3502 in each site.**

Description: He-values are shown for m1501 and m3502 separately, and both loci combined where possible.

|  | 1501 | 3502 | 1501-3502 |
| --- | --- | --- | --- |
| Colombia | 0.70 | 0.76 |  |
| Ecuador | 0.22 | 0.76 | 0.78 |
| Venezuela | 0.73 | 0.57 | 0.84 |
| São Tomé | 0.83 | 0.83 | 1.0 |
| Sudan | 0.70 | 1.0 | 1.0 |
| Pakistan | 0.91 | 0.80 | 0.98 |
| India | 0.90 | 0.86 |  |
| Sri Lanka | 0.85 | 0.74 | 0.92 |
| Nepal | 0.94 | 0.80 | 0.98 |
| Laos | 0.83 | 0.90 |  |
| Thailand | 0.89 | 0.85 |  |
| Korea | 0.42 | 0.73 | 0.81 |
| PNG | 0.86 | 0.86 |  |
