# Supplementary material for: Global and local genetic diversity at two microsatellite loci in Plasmodium vivax parasites from Asia, Africa and South America
Source: Malar J. 2014 Oct 2;13:392. doi: 10.1186/1475-2875-13-392 (PMC4200131; doi:10.1186/1475-2875-13-392)
Supplement: Supplementary file 7 — Additional file 7: Global analysis of molecular variance (AMOVA) among P. vivax populations and continents (groups). (DOCX 16 KB) [file 12936_2014_3558_MOESM7_ESM.docx]

**Additional file 7**

Title: **Global analysis of molecular variance (AMOVA) among *P. vivax* populations and continents (groups**).

Description: ^a^ Columbia, Ecuador, Venezuela, India, Korea, Laos, Nepal, Pakistan, Sri Lanka, Thailand, São Tomé and Sudan.

^b^ Ecuador, Venezuela, Nepal, Pakistan, Sri Lanka, São Tomé, and Sudan.

^c^ All the countries from the 12 included populations (^a^) grouped together.

^d^ 3 continents: Asia (India, Korea, Laos, Nepal, Pakistan, Sri Lanka, Thailand), The Americas (Columbia, Ecuador, Venezuela) and Africa (São Tomé , Sudan).

| Source of variation | Global AMOVA for *P. vivax* | | | | | |
| --- | --- | --- | --- | --- | --- | --- |
|  | m1501 | | m3502 | | 2 loci | |
| Within populations (%) | 89.61 | 86.17 | 89.55 | 86.81 | 88.43 | 92.53 |
| Among populations within groups (%) | 10.39 | 7.88 | 10.45 | 8.54 | 11.57 | 4.20 |
| Among groups (%) | --- | 5.95 | --- | 4.66 | --- | 3.27 |
| Number of populations | 12^a^ | 12^a^ | 12^a^ | 12^a^ | 7^b^ | 7^b^ |
| Number of groups | 1^c^ | 3^d^ | 1^c^ | 3^d^ | 1^c^ | 3^d^ |
| n = | 1248 | | 1226 | | 814 | |
